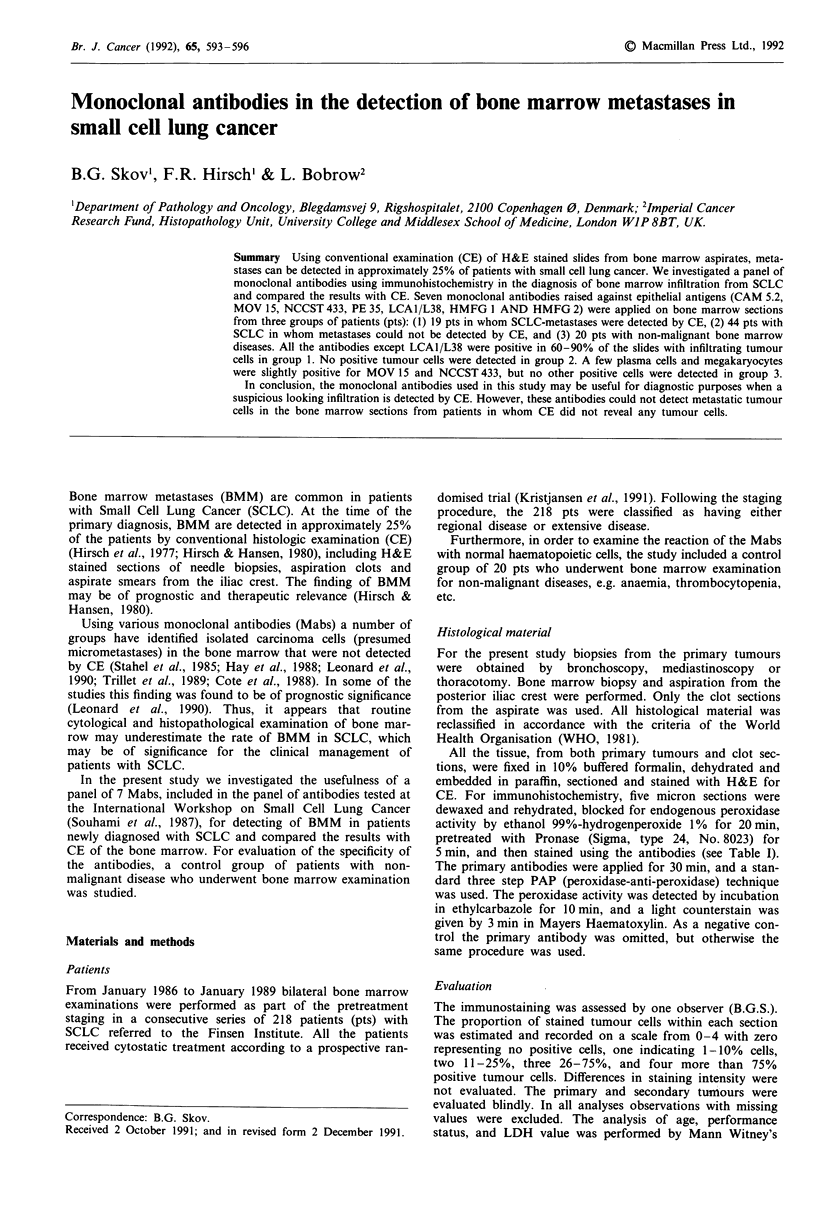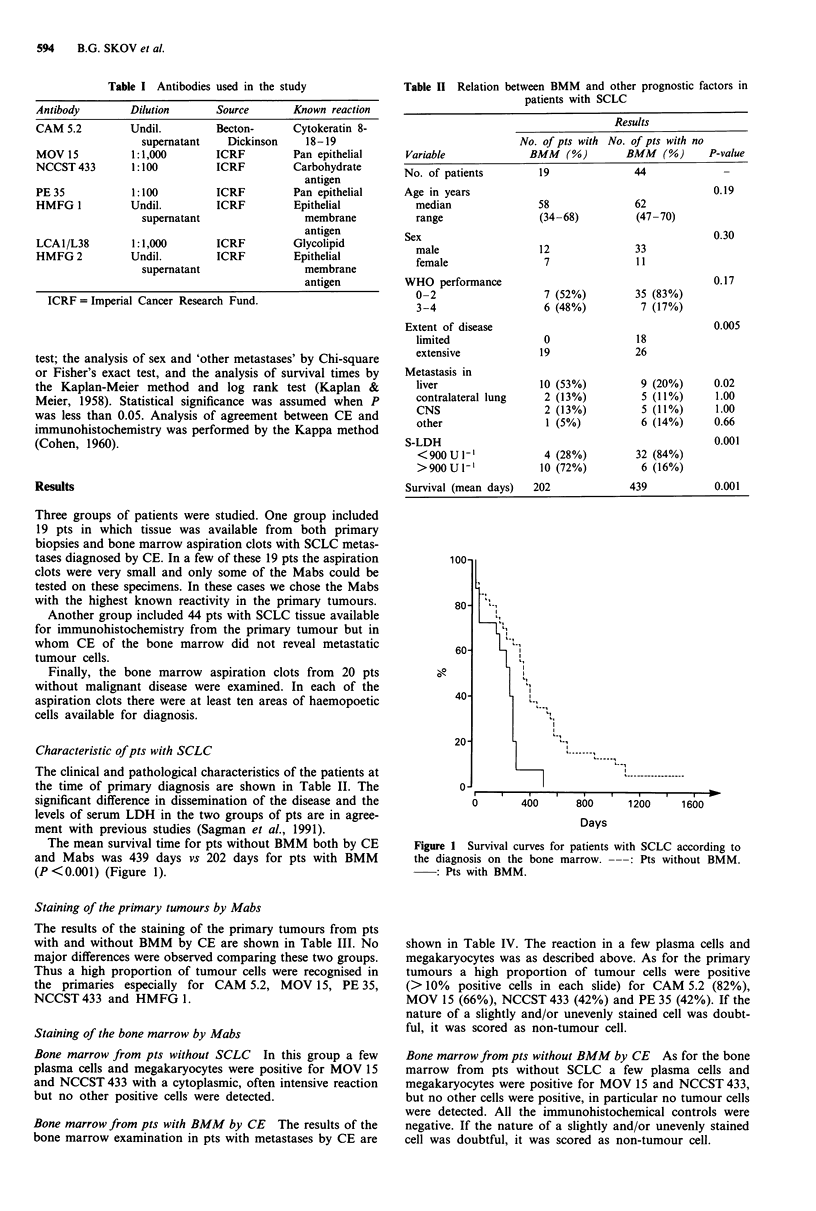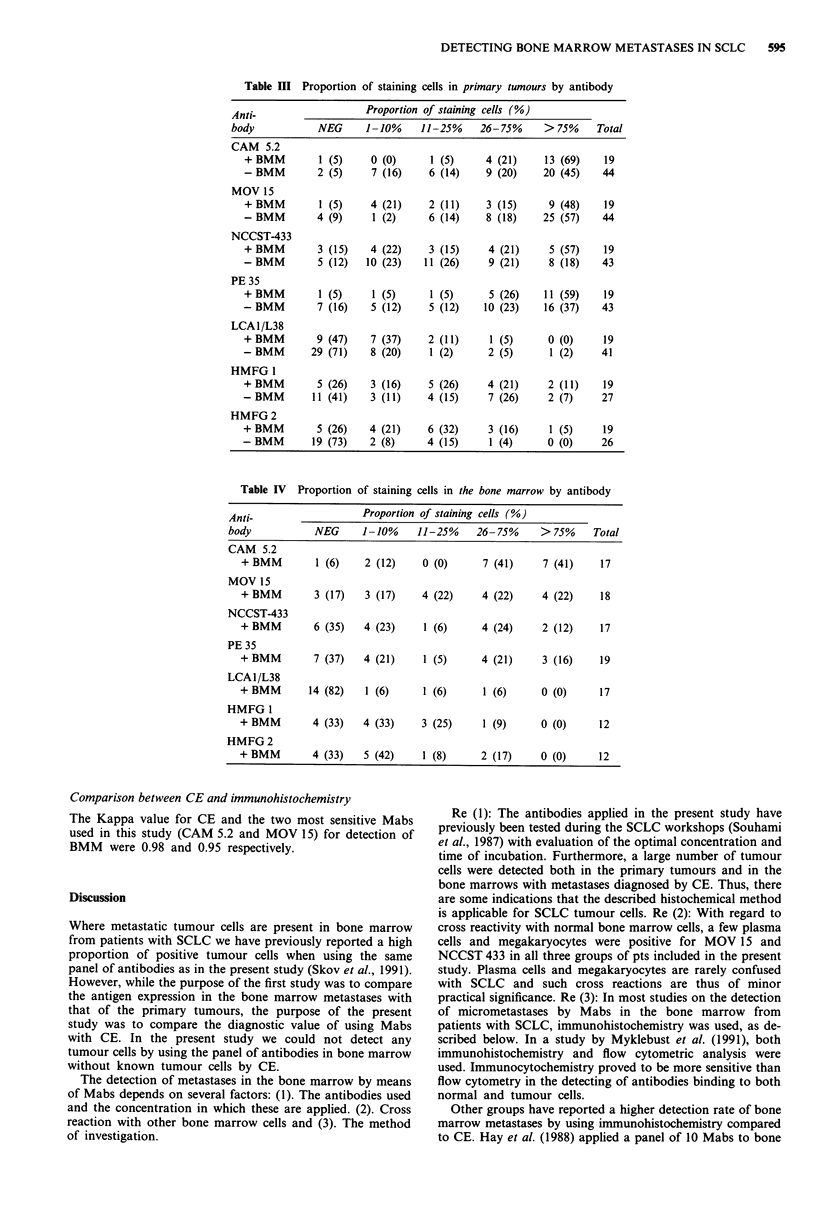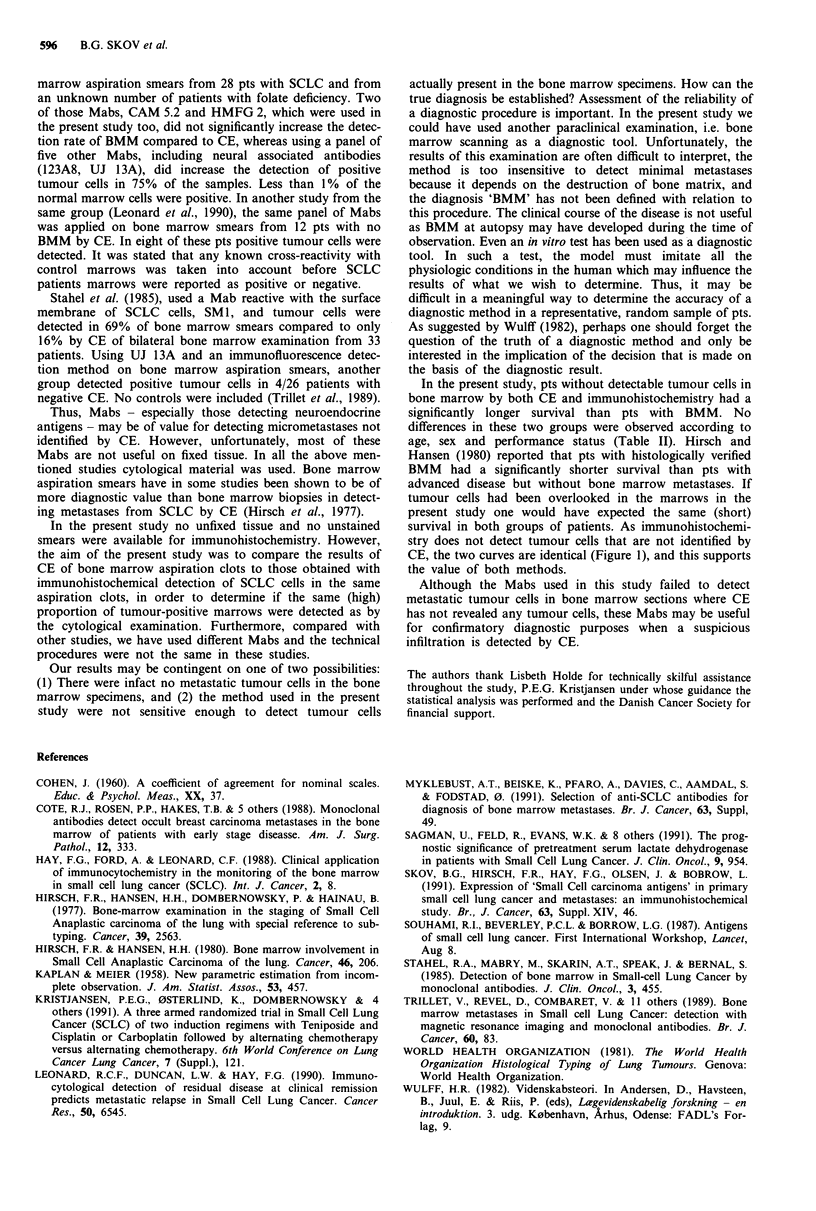# Monoclonal antibodies in the detection of bone marrow metastases in small cell lung cancer.

**DOI:** 10.1038/bjc.1992.120

**Published:** 1992-04

**Authors:** B. G. Skov, F. R. Hirsch, L. Bobrow

**Affiliations:** Department of Pathology, Rigshospitalet, Copenhagen, Denmark.

## Abstract

Using conventional examination (CE) of H&E stained slides from bone marrow aspirates, metastases can be detected in approximately 25% of patients with small cell lung cancer. We investigated a panel of monoclonal antibodies using immunohistochemistry in the diagnosis of bone marrow infiltration from SCLC and compared the results with CE. Seven monoclonal antibodies raised against epithelial antigens (CAM 5.2, MOV 15, NCCST 433, PE 35, LCA1/L38, HMFG 1 AND HMFG 2) were applied on bone marrow sections from three groups of patients (pts): (1) 19 pts in whom SCLC-metastases were detected by CE, (2) 44 pts with SCLC in whom metastases could not be detected by CE, and (3) 20 pts with non-malignant bone marrow diseases. All the antibodies except LCA1/L38 were positive in 60-90% of the slides with infiltrating tumour cells in group 1. No positive tumour cells were detected in group 2. A few plasma cells and megakaryocytes were slightly positive for MOV 15 and NCCST 433, but no other positive cells were detected in group 3. In conclusion, the monoclonal antibodies used in this study may be useful for diagnostic purposes when a suspicious looking infiltration is detected by CE. However, these antibodies could not detect metastatic tumour cells in the bone marrow sections from patients in whom CE did not reveal any tumour cells.


Br. J. Cancer (1992), 65, 593-596 © Macmillan Press Ltd., 1992

### Monoclonal antibodies in the detection of bone marrow metastases in small cell lung cancer

B.G. Skov[1], F.R. Hirsch[1] & L. Bobrow[2]

[1]*Department of Pathology and Oncology, Blegdamsvej 9, Rigshospitalet, 2100 Copenhagen Ø, Denmark;* [2]*Imperial Cancer Research Fund, Histopathology Unit, University College and Middlesex School of Medicine, London W1P 8BT, UK.*

**Summary** Using conventional examination (CE) of H&E stained slides from bone marrow aspirates, metastases can be detected in approximately 25% of patients with small cell lung cancer. We investigated a panel of monoclonal antibodies using immunohistochemistry in the diagnosis of bone marrow infiltration from SCLC and compared the results with CE. Seven monoclonal antibodies raised against epithelial antigens (CAM 5.2, MOV 15, NCCST 433, PE 35, LCA1/L38, HMFG 1 AND HMFG 2) were applied on bone marrow sections from three groups of patients (pts): (1) 19 pts in whom SCLC-metastases were detected by CE, (2) 44 pts with SCLC in whom metastases could not be detected by CE, and (3) 20 pts with non-malignant bone marrow diseases. All the antibodies except LCA1/L38 were positive in 60–90% of the slides with infiltrating tumour cells in group 1. No positive tumour cells were detected in group 2. A few plasma cells and megakaryocytes were slightly positive for MOV 15 and NCCST 433, but no other positive cells were detected in group 3.

In conclusion, the monoclonal antibodies used in this study may be useful for diagnostic purposes when a suspicious looking infiltration is detected by CE. However, these antibodies could not detect metastatic tumour cells in the bone marrow sections from patients in whom CE did not reveal any tumour cells.

Bone marrow metastases (BMM) are common in patients with Small Cell Lung Cancer (SCLC). At the time of the primary diagnosis, BMM are detected in approximately 25% of the patients by conventional histologic examination (CE) (Hirsch *et al.*, 1977; Hirsch & Hansen, 1980), including H&E stained sections of needle biopsies, aspiration clots and aspirate smears from the iliac crest. The finding of BMM may be of prognostic and therapeutic relevance (Hirsch & Hansen, 1980).

Using various monoclonal antibodies (Mabs) a number of groups have identified isolated carcinoma cells (presumed micrometastases) in the bone marrow that were not detected by CE (Stahel *et al.*, 1985; Hay *et al.*, 1988; Leonard *et al.*, 1990; Trillet *et al.*, 1989; Cote *et al.*, 1988). In some of the studies this finding was found to be of prognostic significance (Leonard *et al.*, 1990). Thus, it appears that routine cytological and histopathological examination of bone marrow may underestimate the rate of BMM in SCLC, which may be of significance for the clinical management of patients with SCLC.

In the present study we investigated the usefulness of a panel of 7 Mabs, included in the panel of antibodies tested at the International Workshop on Small Cell Lung Cancer (Souhami *et al.*, 1987), for detecting of BMM in patients newly diagnosed with SCLC and compared the results with CE of the bone marrow. For evaluation of the specificity of the antibodies, a control group of patients with non-malignant disease who underwent bone marrow examination was studied.

### Materials and methods

### *Patients*

From January 1986 to January 1989 bilateral bone marrow examinations were performed as part of the pretreatment staging in a consecutive series of 218 patients (pts) with SCLC referred to the Finsen Institute. All the patients received cytostatic treatment according to a prospective randomised trial (Kristjansen *et al.*, 1991). Following the staging procedure, the 218 pts were classified as having either regional disease or extensive disease.

Furthermore, in order to examine the reaction of the Mabs with normal haematopoietic cells, the study included a control group of 20 pts who underwent bone marrow examination for non-malignant diseases, e.g. anaemia, thrombocytopenia, etc.

### *Histological material*

For the present study biopsies from the primary tumours were obtained by bronchoscopy, mediastinoscopy or thoracotomy. Bone marrow biopsy and aspiration from the posterior iliac crest were performed. Only the clot sections from the aspirate was used. All histological material was reclassified in accordance with the criteria of the World Health Organisation (WHO, 1981).

All the tissue, from both primary tumours and clot sections, were fixed in 10% buffered formalin, dehydrated and embedded in paraffin, sectioned and stained with H&E for CE. For immunohistochemistry, five micron sections were dewaxed and rehydrated, blocked for endogenous peroxidase activity by ethanol 99%-hydrogenperoxide 1% for 20 min, pretreated with Pronase (Sigma, type 24, No. 8023) for 5 min, and then stained using the antibodies (see Table I). The primary antibodies were applied for 30 min, and a standard three step PAP (peroxidase-anti-peroxidase) technique was used. The peroxidase activity was detected by incubation in ethylcarbazole for 10 min, and a light counterstain was given by 3 min in Mayers Haematoxylin. As a negative control the primary antibody was omitted, but otherwise the same procedure was used.

### *Evaluation*

The immunostaining was assessed by one observer (B.G.S.). The proportion of stained tumour cells within each section was estimated and recorded on a scale from 0–4 with zero representing no positive cells, one indicating 1–10% cells, two 11–25%, three 26–75%, and four more than 75% positive tumour cells. Differences in staining intensity were not evaluated. The primary and secondary tumours were evaluated blindly. In all analyses observations with missing values were excluded. The analysis of age, performance status, and LDH value was performed by Mann Witney's

Correspondence: B.G. Skov.
Received 2 October 1991; and in revised form 2 December 1991.

**Table I** Antibodies used in the study

| *Antibody* | *Dilution* | *Source* | *Known reaction* |
|---|---|---|---|
| CAM 5.2 | Undil. supernatant | Becton-Dickinson | Cytokeratin 8-18-19 |
| MOV 15 | 1:1,000 | ICRF | Pan epithelial |
| NCCST 433 | 1:100 | ICRF | Carbohydrate antigen |
| PE 35 | 1:100 | ICRF | Pan epithelial |
| HMFG 1 | Undil. supernatant | ICRF | Epithelial membrane antigen |
| LCA1/L38 | 1:1,000 | ICRF | Glycolipid |
| HMFG 2 | Undil. supernatant | ICRF | Epithelial membrane antigen |

ICRF = Imperial Cancer Research Fund.

test; the analysis of sex and 'other metastases' by Chi-square or Fisher's exact test, and the analysis of survival times by the Kaplan-Meier method and log rank test (Kaplan & Meier, 1958). Statistical significance was assumed when $P$ was less than 0.05. Analysis of agreement between CE and immunohistochemistry was performed by the Kappa method (Cohen, 1960).

### Results

Three groups of patients were studied. One group included 19 pts in which tissue was available from both primary biopsies and bone marrow aspiration clots with SCLC metastases diagnosed by CE. In a few of these 19 pts the aspiration clots were very small and only some of the Mabs could be tested on these specimens. In these cases we chose the Mabs with the highest known reactivity in the primary tumours.

Another group included 44 pts with SCLC tissue available for immunohistochemistry from the primary tumour but in whom CE of the bone marrow did not reveal metastatic tumour cells.

Finally, the bone marrow aspiration clots from 20 pts without malignant disease were examined. In each of the aspiration clots there were at least ten areas of haemopoetic cells available for diagnosis.

### *Characteristic of pts with SCLC*

The clinical and pathological characteristics of the patients at the time of primary diagnosis are shown in Table II. The significant difference in dissemination of the disease and the levels of serum LDH in the two groups of pts are in agreement with previous studies (Sagman *et al.*, 1991).

The mean survival time for pts without BMM both by CE and Mabs was 439 days *vs* 202 days for pts with BMM ($P < 0.001$) (Figure 1).

### *Staining of the primary tumours by Mabs*

The results of the staining of the primary tumours from pts with and without BMM by CE are shown in Table III. No major differences were observed comparing these two groups. Thus a high proportion of tumour cells were recognised in the primaries especially for CAM 5.2, MOV 15, PE 35, NCCST 433 and HMFG 1.

### *Staining of the bone marrow by Mabs*

*Bone marrow from pts without SCLC* In this group a few plasma cells and megakaryocytes were positive for MOV 15 and NCCST 433 with a cytoplasmic, often intensive reaction but no other positive cells were detected.

*Bone marrow from pts with BMM by CE* The results of the bone marrow examination in pts with metastases by CE are shown in Table IV. The reaction in a few plasma cells and megakaryocytes was as described above. As for the primary tumours a high proportion of tumour cells were positive (>10% positive cells in each slide) for CAM 5.2 (82%), MOV 15 (66%), NCCST 433 (42%) and PE 35 (42%). If the nature of a slightly and/or unevenly stained cell was doubtful, it was scored as non-tumour cell.

**Table II** Relation between BMM and other prognostic factors in patients with SCLC

| *Variable* | *Results* No. of pts with BMM (%) | No. of pts with no BMM (%) | *P-value* |
|---|---|---|---|
| No. of patients | 19 | 44 | – |
| Age in years | | | 0.19 |
| median | 58 | 62 | |
| range | (34–68) | (47–70) | |
| Sex | | | 0.30 |
| male | 12 | 33 | |
| female | 7 | 11 | |
| WHO performance | | | 0.17 |
| 0–2 | 7 (52%) | 35 (83%) | |
| 3–4 | 6 (48%) | 7 (17%) | |
| Extent of disease | | | 0.005 |
| limited | 0 | 18 | |
| extensive | 19 | 26 | |
| Metastasis in | | | |
| liver | 10 (53%) | 9 (20%) | 0.02 |
| contralateral lung | 2 (13%) | 5 (11%) | 1.00 |
| CNS | 2 (13%) | 5 (11%) | 1.00 |
| other | 1 (5%) | 6 (14%) | 0.66 |
| S-LDH | | | 0.001 |
| <900 U l$^{-1}$ | 4 (28%) | 32 (84%) | |
| >900 U l$^{-1}$ | 10 (72%) | 6 (16%) | |
| Survival (mean days) | 202 | 439 | 0.001 |

**Figure 1** Survival curves for patients with SCLC according to the diagnosis on the bone marrow. ---: Pts without BMM. ——: Pts with BMM.

*Bone marrow from pts without BMM by CE* As for the bone marrow from pts without SCLC a few plasma cells and megakaryocytes were positive for MOV 15 and NCCST 433, but no other cells were positive, in particular no tumour cells were detected. All the immunohistochemical controls were negative. If the nature of a slightly and/or unevenly stained cell was doubtful, it was scored as non-tumour cell.

**Table III** Proportion of staining cells in *primary tumours* by antibody

| *Anti-body* | *Proportion of staining cells (%)* NEG | *1–10%* | *11–25%* | *26–75%* | *>75%* | *Total* |
|---|---|---|---|---|---|---|
| CAM 5.2 | | | | | | |
| + BMM | 1 (5) | 0 (0) | 1 (5) | 4 (21) | 13 (69) | 19 |
| − BMM | 2 (5) | 7 (16) | 6 (14) | 9 (20) | 20 (45) | 44 |
| MOV 15 | | | | | | |
| + BMM | 1 (5) | 4 (21) | 2 (11) | 3 (15) | 9 (48) | 19 |
| − BMM | 4 (9) | 1 (2) | 6 (14) | 8 (18) | 25 (57) | 44 |
| NCCST-433 | | | | | | |
| + BMM | 3 (15) | 4 (22) | 3 (15) | 4 (21) | 5 (57) | 19 |
| − BMM | 5 (12) | 10 (23) | 11 (26) | 9 (21) | 8 (18) | 43 |
| PE 35 | | | | | | |
| + BMM | 1 (5) | 1 (5) | 1 (5) | 5 (26) | 11 (59) | 19 |
| − BMM | 7 (16) | 5 (12) | 5 (12) | 10 (23) | 16 (37) | 43 |
| LCA1/L38 | | | | | | |
| + BMM | 9 (47) | 7 (37) | 2 (11) | 1 (5) | 0 (0) | 19 |
| − BMM | 29 (71) | 8 (20) | 1 (2) | 2 (5) | 1 (2) | 41 |
| HMFG 1 | | | | | | |
| + BMM | 5 (26) | 3 (16) | 5 (26) | 4 (21) | 2 (11) | 19 |
| − BMM | 11 (41) | 3 (11) | 4 (15) | 7 (26) | 2 (7) | 27 |
| HMFG 2 | | | | | | |
| + BMM | 5 (26) | 4 (21) | 6 (32) | 3 (16) | 1 (5) | 19 |
| − BMM | 19 (73) | 2 (8) | 4 (15) | 1 (4) | 0 (0) | 26 |

**Table IV** Proportion of staining cells in *the bone marrow* by antibody

| *Anti-body* | *Proportion of staining cells (%)* NEG | *1–10%* | *11–25%* | *26–75%* | *>75%* | *Total* |
|---|---|---|---|---|---|---|
| CAM 5.2 | | | | | | |
| + BMM | 1 (6) | 2 (12) | 0 (0) | 7 (41) | 7 (41) | 17 |
| MOV 15 | | | | | | |
| + BMM | 3 (17) | 3 (17) | 4 (22) | 4 (22) | 4 (22) | 18 |
| NCCST-433 | | | | | | |
| + BMM | 6 (35) | 4 (23) | 1 (6) | 4 (24) | 2 (12) | 17 |
| PE 35 | | | | | | |
| + BMM | 7 (37) | 4 (21) | 1 (5) | 4 (21) | 3 (16) | 19 |
| LCA1/L38 | | | | | | |
| + BMM | 14 (82) | 1 (6) | 1 (6) | 1 (6) | 0 (0) | 17 |
| HMFG 1 | | | | | | |
| + BMM | 4 (33) | 4 (33) | 3 (25) | 1 (9) | 0 (0) | 12 |
| HMFG 2 | | | | | | |
| + BMM | 4 (33) | 5 (42) | 1 (8) | 2 (17) | 0 (0) | 12 |

*Comparison between CE and immunohistochemistry*

The Kappa value for CE and the two most sensitive Mabs used in this study (CAM 5.2 and MOV 15) for detection of BMM were 0.98 and 0.95 respectively.

### Discussion

Where metastatic tumour cells are present in bone marrow from patients with SCLC we have previously reported a high proportion of positive tumour cells when using the same panel of antibodies as in the present study (Skov *et al.*, 1991). However, while the purpose of the first study was to compare the antigen expression in the bone marrow metastases with that of the primary tumours, the purpose of the present study was to compare the diagnostic value of using Mabs with CE. In the present study we could not detect any tumour cells by using the panel of antibodies in bone marrow without known tumour cells by CE.

The detection of metastases in the bone marrow by means of Mabs depends on several factors: (1). The antibodies used and the concentration in which these are applied. (2). Cross reaction with other bone marrow cells and (3). The method of investigation.

Re (1): The antibodies applied in the present study have previously been tested during the SCLC workshops (Souhami *et al.*, 1987) with evaluation of the optimal concentration and time of incubation. Furthermore, a large number of tumour cells were detected both in the primary tumours and in the bone marrows with metastases diagnosed by CE. Thus, there are some indications that the described histochemical method is applicable for SCLC tumour cells. Re (2): With regard to cross reactivity with normal bone marrow cells, a few plasma cells and megakaryocytes were positive for MOV 15 and NCCST 433 in all three groups of pts included in the present study. Plasma cells and megakaryocytes are rarely confused with SCLC and such cross reactions are thus of minor practical significance. Re (3): In most studies on the detection of micrometastases by Mabs in the bone marrow from patients with SCLC, immunohistochemistry was used, as described below. In a study by Myklebust *et al.* (1991), both immunohistochemistry and flow cytometric analysis were used. Immunocytochemistry proved to be more sensitive than flow cytometry in the detecting of antibodies binding to both normal and tumour cells.

Other groups have reported a higher detection rate of bone marrow metastases by using immunohistochemistry compared to CE. Hay *et al.* (1988) applied a panel of 10 Mabs to bone marrow aspiration smears from 28 pts with SCLC and from an unknown number of patients with folate deficiency. Two of those Mabs, CAM 5.2 and HMFG 2, which were used in the present study too, did not significantly increase the detection rate of BMM compared to CE, whereas using a panel of five other Mabs, including neural associated antibodies (123A8, UJ 13A), did increase the detection of positive tumour cells in 75% of the samples. Less than 1% of the normal marrow cells were positive. In another study from the same group (Leonard *et al.*, 1990), the same panel of Mabs was applied on bone marrow smears from 12 pts with no BMM by CE. In eight of these pts positive tumour cells were detected. It was stated that any known cross-reactivity with control marrows was taken into account before SCLC patients marrows were reported as positive or negative.

Stahel *et al.* (1985), used a Mab reactive with the surface membrane of SCLC cells, SM1, and tumour cells were detected in 69% of bone marrow smears compared to only 16% by CE of bilateral bone marrow examination from 33 patients. Using UJ 13A and an immunofluorescence detection method on bone marrow aspiration smears, another group detected positive tumour cells in 4/26 patients with negative CE. No controls were included (Trillet *et al.*, 1989).

Thus, Mabs – especially those detecting neuroendocrine antigens – may be of value for detecting micrometastases not identified by CE. However, unfortunately, most of these Mabs are not useful on fixed tissue. In all the above mentioned studies cytological material was used. Bone marrow aspiration smears have in some studies been shown to be of more diagnostic value than bone marrow biopsies in detecting metastases from SCLC by CE (Hirsch *et al.*, 1977).

In the present study no unfixed tissue and no unstained smears were available for immunohistochemistry. However, the aim of the present study was to compare the results of CE of bone marrow aspiration clots to those obtained with immunohistochemical detection of SCLC cells in the same aspiration clots, in order to determine if the same (high) proportion of tumour-positive marrows were detected as by the cytological examination. Furthermore, compared with other studies, we have used different Mabs and the technical procedures were not the same in these studies.

Our results may be contingent on one of two possibilities: (1) There were infact no metastatic tumour cells in the bone marrow specimens, and (2) the method used in the present study were not sensitive enough to detect tumour cells actually present in the bone marrow specimens. How can the true diagnosis be established? Assessment of the reliability of a diagnostic procedure is important. In the present study we could have used another paraclinical examination, i.e. bone marrow scanning as a diagnostic tool. Unfortunately, the results of this examination are often difficult to interpret, the method is too insensitive to detect minimal metastases because it depends on the destruction of bone matrix, and the diagnosis 'BMM' has not been defined with relation to this procedure. The clinical course of the disease is not useful as BMM at autopsy may have developed during the time of observation. Even an *in vitro* test has been used as a diagnostic tool. In such a test, the model must imitate all the physiologic conditions in the human which may influence the results of what we wish to determine. Thus, it may be difficult in a meaningful way to determine the accuracy of a diagnostic method in a representative, random sample of pts. As suggested by Wulff (1982), perhaps one should forget the question of the truth of a diagnostic method and only be interested in the implication of the decision that is made on the basis of the diagnostic result.

In the present study, pts without detectable tumour cells in bone marrow by both CE and immunohistochemistry had a significantly longer survival than pts with BMM. No differences in these two groups were observed according to age, sex and performance status (Table II). Hirsch and Hansen (1980) reported that pts with histologically verified BMM had a significantly shorter survival than pts with advanced disease but without bone marrow metastases. If tumour cells had been overlooked in the marrows in the present study one would have expected the same (short) survival in both groups of patients. As immunohistochemistry does not detect tumour cells that are not identified by CE, the two curves are identical (Figure 1), and this supports the value of both methods.

Although the Mabs used in this study failed to detect metastatic tumour cells in bone marrow sections where CE has not revealed any tumour cells, these Mabs may be useful for confirmatory diagnostic purposes when a suspicious infiltration is detected by CE.

The authors thank Lisbeth Holde for technically skilful assistance throughout the study, P.E.G. Kristjansen under whose guidance the statistical analysis was performed and the Danish Cancer Society for financial support.

### References

COHEN, J. (1960). A coefficient of agreement for nominal scales. *Educ. & Psychol. Meas.*, **XX**, 37.

COTE, R.J., ROSEN, P.P., HAKES, T.B. & 5 others (1988). Monoclonal antibodies detect occult breast carcinoma metastases in the bone marrow of patients with early stage diseasse. *Am. J. Surg. Pathol.*, **12**, 333.

HAY, F.G., FORD, A. & LEONARD, C.F. (1988). Clinical application of immunocytochemistry in the monitoring of the bone marrow in small cell lung cancer (SCLC). *Int. J. Cancer*, **2**, 8.

HIRSCH, F.R., HANSEN, H.H., DOMBERNOWSKY, P. & HAINAU, B. (1977). Bone-marrow examination in the staging of Small Cell Anaplastic carcinoma of the lung with special reference to subtyping. *Cancer*, **39**, 2563.

HIRSCH, F.R. & HANSEN, H.H. (1980). Bone marrow involvement in Small Cell Anaplastic Carcinoma of the lung. *Cancer*, **46**, 206.

KAPLAN & MEIER (1958). New parametric estimation from incomplete observation. *J. Am. Statist. Assos.*, **53**, 457.

KRISTJANSEN, P.E.G., ØSTERLIND, K., DOMBERNOWSKY & 4 others (1991). A three armed randomized trial in Small Cell Lung Cancer (SCLC) of two induction regimens with Teniposide and Cisplatin or Carboplatin followed by alternating chemotherapy versus alternating chemotherapy. *6th World Conference on Lung Cancer Lung Cancer*, **7** (Suppl.), 121.

LEONARD, R.C.F., DUNCAN, L.W. & HAY, F.G. (1990). Immunocytological detection of residual disease at clinical remission predicts metastatic relapse in Small Cell Lung Cancer. *Cancer Res.*, **50**, 6545.

MYKLEBUST, A.T., BEISKE, K., PFARO, A., DAVIES, C., AAMDAL, S. & FODSTAD, Ø. (1991). Selection of anti-SCLC antibodies for diagnosis of bone marrow metastases. *Br. J. Cancer*, **63**, Suppl, 49.

SAGMAN, U., FELD, R., EVANS, W.K. & 8 others (1991). The prognostic significance of pretreatment serum lactate dehydrogenase in patients with Small Cell Lung Cancer. *J. Clin. Oncol.*, **9**, 954.

SKOV, B.G., HIRSCH, F.R., HAY, F.G., OLSEN, J. & BOBROW, L. (1991). Expression of 'Small Cell carcinoma antigens' in primary small cell lung cancer and metastases: an immunohistochemical study. *Br., J. Cancer*, **63**, Suppl. XIV, 46.

SOUHAMI, R.I., BEVERLEY, P.C.L. & BORROW, L.G. (1987). Antigens of small cell lung cancer. First International Workshop, *Lancet*, Aug 8.

STAHEL, R.A., MABRY, M., SKARIN, A.T., SPEAK, J. & BERNAL, S. (1985). Detection of bone marrow in Small-cell Lung Cancer by monoclonal antibodies. *J. Clin. Oncol.*, **3**, 455.

TRILLET, V., REVEL, D., COMBARET, V. & 11 others (1989). Bone marrow metastases in Small cell Lung Cancer: detection with magnetic resonance imaging and monoclonal antibodies. *Br. J. Cancer*, **60**, 83.

WORLD HEALTH ORGANIZATION (1981). *The World Health Organization Histological Typing of Lung Tumours*. Genova: World Health Organization.

WULFF, H.R. (1982). Videnskabsteori. In Andersen, D., Havsteen, B., Juul, E. & Riis, P. (eds), *Lægevidenskabelig forskning – en introduktion*. 3. udg. København, Århus, Odense: FADL's Forlag, 9.